# Cross-cultural adaptation and validation of the simplified Chinese version of the functional assessment scale for acute hamstring injuries (FASH) questionnaire

**DOI:** 10.1186/s13018-025-06578-2

**Published:** 2025-12-31

**Authors:** Yu-Jie Wu, Ning Liu, Qiang Zheng, Kan Liu, Qing-Meng Zhang, Shi-Qi Cao

**Affiliations:** 1 Department of Nursing, The Third People’s Hospital of Datong, Shanxi, China; 2 Department of Orthopedics, The 960th Hospital of Joint Logistics Support Force, Jinan, Shandong China; 3 Department of Emergency Surgery and Orthopaedic Surgery, Qilu Hospital of Shandong University, Jinan, 250012 Shandong China; 4 Department of Orthopedics, Beijing University of Chinese Medicine Third Affiliated Hospital, Beijing, 100029 People’s Republic of China; 5 Department of Orthopaedics, Qilu Hospital of Shandong University, Jinan, Shandong 250012 People’s Republic of China; 6 Orthopaedics of TCM Senior Department, The Sixth Medical Center of Chinese PLA General Hospital, Beijing, 100048 People’s Republic of China

**Keywords:** Hamstring Injuries, Psychometrics, Chinese, Questionnaire

## Abstract

**Background:**

The Functional Assessment Scale for Acute Hamstring Injuries (FASH) Questionnaire is a tool to assess the severity of symptoms and their impact on function and sports ability in patients with acute hamstring injuries. The study was to cross-culturally adapt and psychometrically validate a Simplified Chinese version of the FASH (SC-FASH).

**Methods:**

Cross-cultural adaptation was performed in accordance with the internationally recognised guidelines of the American Academy of Orthopaedic Surgeons Outcome Committee. The FASH is a 10-item questionnaire. The sample size should be 5 to 10 times the total number of items on the scale. 150 participants with acute hamstring injuries were included in this study. Cronbach's α and the intra-class correlation coefficient (ICC) were used to assess reliability, and correlations between the SC-FASH and the Exercise-Induced Leg Pain Questionnaire (EILP), the Visual Analogue Scale (VAS) and the Short Form (36) Health Survey (SF-36) were used to assess construct validity.

**Results:**

Between February and May 2025, 150 literate native Chinese speakers with hamstring injuries were recruited for a study. All the 10 items had an answer rate of 100%. The FASH was successfully adapted and translated into Simplified Chinese. Each item was appropriately correlated with the total items. Good reliabilities were observed in SC-FASH, evidenced by Cronbach's alpha of 0.88, an ICC 0.97 (95% CI, 0.96–0.98). The elimination of any one item did not result in a Cronbach's α < 0.80. Standard Error of the Mean (SEM) was 4.41. Good correlations were observed with EILP (0.68, *p* < 0.01), VAS (-0.62, *p* < 0.01), and physical function of SF-36 (0.62, *p* < 0.01). Moderate correlations were observed with role physical (0.46, *p* < 0.01), bodily pain (0.50, *p* < 0.01), and general health (0.44, *p* < 0.01). Fair to poor correlations were observed with vitality, social function, role emotional, and mental health domains of SF-36.

**Conclusion:**

SC-FASH is recommended to evaluate the severity of hamstring muscle injuries and their impact on physical function and sports ability in Mainland China due to its good reliability, and validity.

## Introduction

As sporting events such as the Olympic Games, football, and the NBA are a regular occurrence in people's lives, hamstring muscle injuries have become a prevalent soft tissue injury among visiting physicians in China [1–3]. The particular lesion occurs in the posterior thigh, affecting the semimembranosus, semitendinosus, and biceps femoris [4, 5]. This condition impacts individuals of all ages and can result in disability, psychosocial challenges, and economic implications (the expenses incurred by long-term rehabilitation), particularly among young people who engage in regular exercise or sporting activities [6, 7]. Extensive research has provided substantial evidence indicating that factors such as acceleration or deceleration in direction and a history of hamstring strain are associated with an elevated risk of hamstring muscle injury [8, 9]. Moreover, hamstring muscle injuries are challenging to differentiate due to the multifactorial nature of their aetiology [10, 11]. Therefore, recovery after such injuries is not so straightforward [12, 13]. Consequently, there is a necessity to devise valid and specific measurement tools to facilitate a more precise evaluation of not only the severity of the injury but also its progression until the individual is able to resume sporting activities [14, 15].

Health-related quality of life (HRQoL) questionnaires are patient-based tools used to understand disorder severity and find better treatment options [16, 17]. According to Consensus-based Standards for the Selection of Health Measurement Instruments, when using a reliable, valid questionnaire in diverse populations, it is necessary to test its psychometric properties to avoid bias caused by cultural differences [18, 19].

The Functional Assessment Scale for Acute Hamstring Injuries (FASH) was initially developed and validated in 2014 for measuring the severity of hamstring muscle injuries and their impact on physical function and sports ability, and has been developed in English [20], Greek [20], German [21], French [22], Spanish [23] and Persian [24]. The present questionnaire is unique in its focus on hamstring muscle injuries [24].

The aim of this study was to translate, culturally adapt, and psychometrically validate the Simplified Chinese version of the Functional Assessment Scale for Acute Hamstring Injuries (SC-FASH). We assumed that the score of SC-FASH should be in accordance with domains of EILP, VAS, and physical domains of SF-36, but not with mental domains of SF-36.

## Materials and methods

### Patients and data collection

Between February and May 2025, in our outpatient clinic, 150 literate native Chinese speakers with hamstring injuries were recruited for a study. They completed two rounds of questionnaires to evaluate test–retest reliability. Feedback after completing the form was collected via a WeChat mini-program survey. Demographic and clinical details are listed in Table 1. Inclusion criteria: age > 18, independent signing authority, able to read and write Chinese language, and participants with exercise habits. Exclusion criteria: pregnant, previous joint disease, referred pain from the lumbar, surgery history (fracture, osteoporosis, trauma), systemic rheumatic diseases, neurological diseases, psychiatric disorders, other uncontrolled systemic disorders (diabetes, tumour, nephritis) [17, 20]. Participants met Terwee et al.'s standards for internal consistency analysis (100 patients) and floor or ceiling effects analysis (50 patients) [17]. All participants signed informed consent, and the study was approved by our hospital's clinical research ethics committee.

**Table 1:** Demographic and clinical characteristics of the participants

Characteristics	Number or mean ± SD
Age (years)	34.54 ± 10.40
Range	18—65
BMI(kg/m^2^)	23.18 ± 3.94
Gender	Total (*N* = 150)
Female	68
Male	82
Duration of Pain(months)	2.69 ± 1.90
Education level	
Primary school	7
High school	24
University	97
Graduate	22
Questionnaire completion time (seconds)	155.80 ± 102.39

BMI: body mass index

On the first day, individuals reported gender, age, body mass index (BMI), pain duration, and education level (Table 1). Then they completed the SC-FASH, the Chinese version Visual Analogue Scale (VAS) [25], the Chinese version Exercise-Induced Leg Pain Questionnaire (EILP) [26], and the Chinese version Short Form (36) Health Survey (SF-36) [27]. Two weeks later, they repeated the SC-FASH to assess test–retest reliability.

### Translation and cross-cultural adaptation Questionnaires

Translation and cross-cultural adaptation followed the internationally recognized guidelines: forward translation, translation synthesis, backward translation, summarization of prefinal version, and finalization [16, 17]. Researchers then discussed the previous test's issues and developed the final SC-FASH (Table 2). We invited a panel of experts comprising five orthopedic specialists and five rehabilitation specialists to evaluate the relevance of the 10 items in the SC-FASH. The composition and qualifications of the expert panel were proved by our hospital (a Grade A tertiary hospital). Calculations revealed that the content validity index at the item level was 1.0, and the content validity index at the scale level was also 1.0, indicating that the scale possesses excellent content validity.

**Table 2:** Steps of translation and trans-cultural adaptation

Steps	Detailed contents
Forward translation	Two bilingual translators independently translated the metric from English to simplified Chinese. One of the translators was an orthopedic surgeon in the author’s hospital; the other one was a professional translator without medical background
Synthesis of the translation	Two translators and other researchers unified contradictions regarding language expression and cultural difference after a consensus meeting and obtained the first SC-FASH
Backward translation	Two native English speakers fluent in English, with medical background and blind to the previous original English version of FASH, independently translated the first SC- FASH back into the English version
Summarization of prefinal version	A consensus meeting with all researchers including four forward and backward translators was held to resolve all discrepancies, ambiguities, or any other verbal issues to reach a prefinal SC- FASH
Determination of final version	Researchers invited 20 patients with hamstring muscle injuries to preliminarily test the prefinal version and collect feedbacks from them

FASH: functional Assessment Scale for Acute Hamstring Injuries; SC: FASH: simplified Chinese Version of the Functional Assessment Scale for Acute Hamstring Injuries

### Questionnaires

The FASH is a disease-specific questionnaire designed to measure individual’s hamstring muscle injuries through different exercise phases and exercise action, including when during walking, during jogging or slow pace running, during accelerating or sprinting for 30 m, during static stretching your hamstrings (toe touch in standing), during functional stretching of your hamstrings (straight leg kick), performing a full weight-bearing lunge, Nordic exercise (partner exercise where you attempt to resist a forward-falling motion using your hamstrings throughout the whole range of motion to the ground), performing 3 one-legged jumps for distance [20].

The EILP is a disease-specific questionnaire measuring an individual’s perception of different exercise phases and exercise action [26]. The VAS is a 10 cm (100 mm) horizontal or vertical self-reported scale anchored at both ends referring to the pain status [25]. The SF-36 assesses general quality of life [27]. All of the aforementioned scales have been translated into Chinese and their reliability and validity have been demonstrated.

### Psychometric assessments

The total score of the SC-FASH is the sum of points from all 10 items, ranging from a minimum of 0 (lowest functional level) to a maximum of 100 (highest functional level). Individuals were asked about the difficulties encountered to assess the acceptability of SC-FASH, and the mean completion time was obtained for all participants (Table 1).

A statistical analysis was conducted to ascertain the distribution of scores [17, 18]. Ceiling and floor effects were considered present if at least 15% of participants assigned the maximum or minimum score [17, 18].

**Fig. 1:**
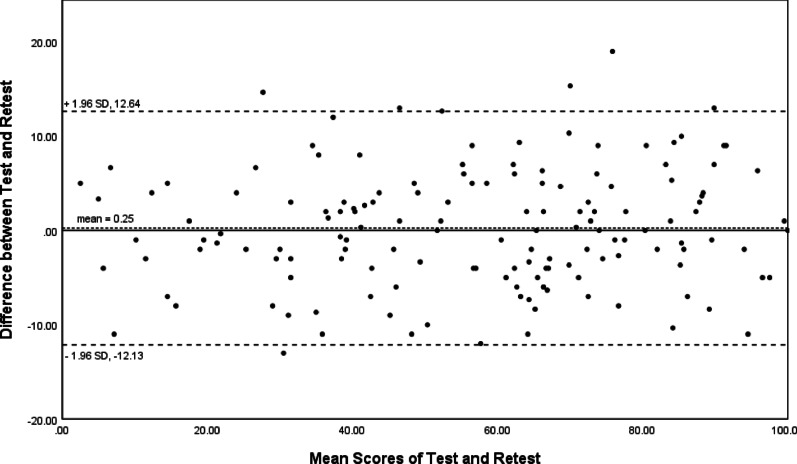
The Bland–Altman plot for test–retest agreement of SC-FASH. The differences between the scores for SC-FASH from the two test sessions were plotted against the mean of the test and retest. The line indicates mean difference value of the two sessions and the 95% (± 1.96 standard deviation) limits of agreement

Reliability included test–retest reliability and internal consistency [17]. Test–retest reliability was assessed by comparing scores from the same individual on two separate occasions. The intra-class correlation coefficient (ICC) was calculated using a two-way analysis. ICC > 0.8 and > 0.9 represented good and excellent reliability. Internal consistency was assessed by Cronbach's alpha. Acceptable, good, and excellent internal consistency was deemed to be > 0.7, 0.8, and 0.9 [17]. Bias between the two measures could be estimated by Bland–Altman plots (Fig. 1). Standard Error of the Mean (SEM): $SEM=\mathrm{SD}\times \sqrt{1-\mathrm{ICC}}$ and Minimum Detectable Change (MDC) ${MDC}_{95}=\mathrm{SEM}\times 1.96\times \sqrt{2}$ were also calculated. SEM indicated the amount of random error in an individual's score, reflecting the precision of the measurement. MDC represented the smallest change in score that was likely to reflect a true change (beyond measurement error). Both metrics helped evaluate the reliability and practical significance of measurement tools.

**Fig. 2:**
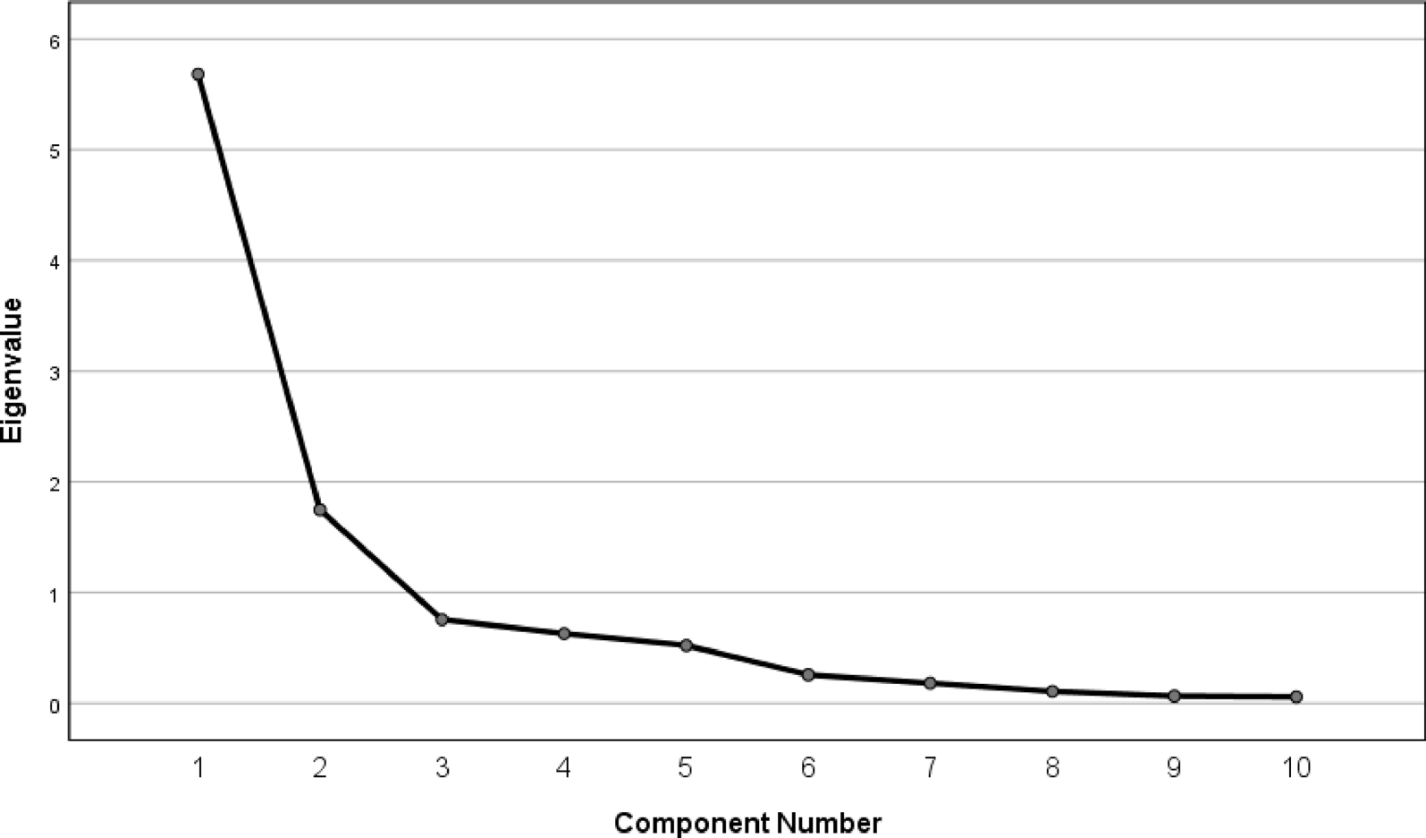
Scree plot of eigenvalues from the 10-item SC-FASH questionnaire

Validity included content validity and construct validity [16, 17]. A rehabilitation expert and three orthopedic experts analyzed the correlation between the content of each item and the disease state to evaluate content validity [28]. Construct validity indicated how well the questionnaire correlated with the same construct (convergent validity), but not with different constructs (divergent or discriminant validity). We assumed that the score of SC-FASH should be in accordance with domains of EILP, VAS, and physical domains of SF-36, but not with mental domains of SF-36. We calculated Pearson's correlation coefficient (r) between SC-FASH and EILP, VAS, and SF-36. Then compared how the data fitted with the calculated correlations to evaluate construct validity as poor (r = 0–0.2), fair (r = 0.2–0.4), moderate (r = 0.4–0.6), very good (r = 0.6–0.8), or excellent (r = 0.8–1.0) [28]. Factorial validity, as part of construct validity, establishes the factor structure of the scale [17, 26]. Exploratory factor analysis (EFA) was used to evaluate the factor structure of the SC-FASH and determine whether its items grouped consistently, providing a more rigorous test than confirmatory factor analysis (CFA) (Fig. 2). Kaiser–Meyer–Olkin (KMO) > 0.6 is acceptable, > 0.8 is good adequacy. Bartlett's Sphericity Test: *p* < 0.001 indicates sufficient correlation among variables, suitable for factor analysis.

### Statistical analysis

SPSS 24.0 was used to analyze the data. Mean values were reported with standard deviation (SD) and ICC values with 95% confidence intervals (CIs). *p* < 0.05 was defined as statistically significant.

## Results

### Translation and cross-cultural adaptation

It was observed that some minor discrepancies emerged in some items due to the influence of cultural diversity. These disparities were addressed through cross-cultural adaptation during the forward and back translations of the FASH. Specifically, each item was scored with a 10-point scale ranging from 0 (unable to perform activity/severe pain/symptoms) to 10 (no difficulty/no pain/symptoms), which was the opposite of the regular VAS rating values. It was easy to make mistakes in filling out the scale. Item 9 “Nordic exercise” was a very professional exercise program. This movement was not a regular exercise in the domestic sports community. We needed to use the picture to explain it and prevent misunderstandings. Item 10 “3 one-legged jumps for distance” was a difficult exercise and required a note on the side for the safety of the tester. Furthermore, a significant number of participants exhibited ambiguity regarding the anatomical location of the acute hamstring injuries, and we needed to dedicate a diagram to explain this in detail to them.

In the pilot trial, three out of ten individuals were confused by the methods of the 'one-legged jump' and 'Nordic exercise'. Under these circumstances, we provided diagrams to explain these exercises in detail and informed all participants of the content verbally. No misunderstandings about the questionnaire arose among subsequent participants.

### Acceptability and score distribution

In the formal study, all participants understood the content of the questionnaire the first time they completed the SC-FASH. All items had an answer rate of 100%.

The absolute values of all the scores were listed in Table 3. No ceiling (2.67%) or floor (1.33%) effects were observed in the total SC-FASH score.

**Table 3:** Absolute values of all scores

Scales	Mean ± SD	Minimum	Median	Maximum
SC-FASH	56.62 ± 24.29	0	62	100
EILP	74.10 ± 25.76	0	80	100
VAS	2.94 ± 3.27	0	2	10
SF-36				
Physical functioning	75.40 ± 28.55	0	85	100
Role physical	70.67 ± 40.83	0	50	100
Bodily pain	80.22 ± 22.05	0	84	100
General health	40.93 ± 15.82	0	40	100
Vitality	46.50 ± 21.46	0	50	100
Social function	49.25 ± 23.09	0	63	100
Role emotional	72.44 ± 39.71	0	67	100
Mental health	47.28 ± 23.79	0	52	100

SC-FASH: simplified Chinese version of the functional assessment scale for acute hamstring injuries; EILP: exercise-induced leg pain questionnaire; VAS: visual analogue scale; SF-36: short form (36) health survey

### Reliability

The Cronbach's α = 0.88, indicated good internal consistency of the SC-FASH. Eliminating one item from each of the ten questions did not result in a value of less than 0.80. All items except questions 2, 9 and 10 correlated with the total score of > 0.74 (Table 4). The ICC = 0.97 (95% CI, 0.96–0.98), indicated good test–retest reliability of the SC-FASH. Bland–Altman plots for the two measures revealed no systematic error (Fig. 1), suggesting good test–retest agreement and reproducibility of the SC-FASH. SEM = 4.41, representing the standard deviation of measurement error. MDC_95_ = 12.22, meaning an individual's score must change by more than 12.22 points to be considered a true change (rather than measurement error), at a 95% confidence level.

**Table 4:** Internal consistency and test–retest reliability of the SC-FASH

Item	Mean ± SD	Item-total correlation	Alpha if item removed
1	6.48 ± 3.33	0.74	0.85
2	4.36 ± 3.78	0.26	0.89
3	7.02 ± 3.59	0.74	0.85
4	6.60 ± 3.49	0.82	0.85
5	6.44 ± 3.40	0.83	0.85
6	5.62 ± 3.23	0.76	0.85
7	5.52 ± 3.17	0.77	0.85
8	6.41 ± 3.19	0.80	0.85
9	3.87 ± 3.81	0.28	0.89
10	4.31 ± 4.26	0.24	0.90

SC-FASH: simplified Chinese version of the functional assessment scale for acute hamstring injuries

### Validity

According to the evaluation by the rehabilitation and orthopedic experts at SC-FASH, good content validities were observed, and the information derived from all the questions was adequate for assessing the impact of acute hamstring injuries on individuals' daily activities. In these circumstances, it was not recommended to add or remove any questions.

Table 5 showed the data of construct validity data for SC-FASH, which were consistent with our consumption. Good correlations were observed with the EILP (0.68, *p* < 0.01), the VAS (-0.62, *p* < 0.01), and the physical function domain of the SF-36 (0.62, *p* < 0.01). Moderate correlations were observed with role physical (0.46, *p* < 0.01), bodily pain (0.50, *p* < 0.01), and general health (0.44, *p* < 0.01). Additionally, fair to poor correlations were observed with vitality, social function, role emotional, and mental health domains of SF-36 (0.14, 0.14, 0.29 and 0.12, respectively).

**Table 5:** Construct validity of the SC-FASH

Scales	Correlation coefficient (r)^a^	*p* value
EILP	.68*	< .001
VAS	-62*	< .001
SF-36		
Physical function	.62*	< .001
Role physical	.46*	< .001
Bodily pain	.50*	< .001
General health	.44*	< .001
Vitality	.14	.08
Social function	.14	.10
Role emotional	.29*	< .001
Mental health	.12	.13

The sample size for the analysis of construct validity was 150

SC-FASH: simplified Chinese version of the functional assessment scale for acute hamstring injuries; EILP: The exercise-induced leg pain questionnaire; VAS: visual analogue scale; SF-36: short form (36) health survey

^*^Correlation is significant at the 0.01 level (two-tailed)

^a^Calculated by the Pearson’s correlation of the SC-FASH with EILP, VAS and SF-36

As stated in the literature, the factor structure of a new version of a questionnaire may differ from the original during the translation and validation process. To investigate this, we ran an analysis to produce a 2-factor solution. We observed that the 2-factor loading explained 74.28% of the total variance (Kaiser–Mayer–Olkin (KMO) = 0.86, C^2^ = 1450.66, *P* < 0.001): Question 1, 3, 4, 5, 6, 7 and 8 for moderate-intensity or regular exercise, Question 2, 9 and 10 for high-intensity or professional exercise. According to the principles of parsimony and interpretability, this model was excluded from further analysis. Bartlett's test of sphericity was significant (*df* = 45, *P* < 0.001) (Fig. 2).

## Discussion

In China, an increasing number of people are taking part in activities such as jogging, football, basketball, tennis, swimming, mountaineering and going to the gym [29, 30]. There was an absence of a rigorous, disease-specific, validated questionnaire that was both comprehensible and designed for use in measuring the severity of symptoms and sporting ability in different movement phases among individuals with acute hamstring muscle injuries, with a view to providing specific therapy options [30–33].

This study constituted the inaugural report on the adaptation of the FASH into Chinese. Other Chinese versions of questionnaires, such as the EILP, VAS and Oliveira, were designed for patients with generalized pain. They may be affected by things like spine disease, joint surgery or other leg pain. However, the FASH questionnaire is utilized for the evaluation of the impact of acute hamstring injuries on daily activities [20, 34, 35]. The questionnaire has been demonstrated to be highly reliable and valid. Consequently, the cross-cultural adaptation of FASH for the Chinese language, the most widely spoken in the world, was deemed to be of significant importance, constituting the fundamental objective of the present study.

In the present study, only minor adjustments were made to certain items in order to account for the cultural differences between China and the West [28]. The English questionnaire underwent no significant changes. This would result in discrepancies between the final two items. Following the adaptation process, it was found that all questions were comprehensible to the participants, and the pretest and formal study revealed that 100% of items were answered. This suggested that the SC-FASH showing good acceptability.

The internal consistency of SC-FASH was good (Cronbach’s alpha = 0.88), but it was lower than that of the original Greek, and English (Cronbach’s alpha = 0.98) [20], the German FASH (Cronbach’s alpha = 0.938) [21], the French FASH (Cronbach’s alpha = 0.969) [22], the Spanish FASH (Cronbach’s alpha = 0.971) [23], and the Persian FASH (Cronbach’s alpha = 0.966) [24]. This was mainly because the purpose of our scale was to target healthy people, rather than subdivided into patient group, athlete group or soccer group, etc. as they are [20, 36, 37]. With the exception of questions 1 and 3, which demonstrated equivalent performance metrics to those of other studies, questions 4, 5, 6, 7 and 8 were scored higher than other studies because these questions were easier for our subjects to complete. All items correlated with the total score of > 0.74 except for questions 2, 9, and 10 (Table 4). The item total correlation of the second question was relatively small because it asked, "Are you currently taking part in your sport, training, or other physical activity?" Most of the participants were relatively healthy people who did not have a specialized training programme, but rather did low-intensity exercises such as walking. This was why the score was also relatively low. For the ninth and tenth question, “Nordic exercise” and “one-legged jumps for distance” were both professional sports involving high intensity, which would be considered high-load exercise for the relatively healthy people included in this study. Consequently, the completion rate was low and the score was also relatively low. Moreover, the majority of individuals in China demonstrated a preference for sporting activities such as square dancing, road walking, cycling, and physical training in sports facilities, which exhibited significant variation both among individuals and between countries in the contemporary globalized Chinese context [26, 28, 38, 39].

Furthermore, the test–retest data was excellent reliability (ICC = 0.97), which was similar to the original Greek, and English FASH (ICC = 0.9) [20], the German FASH (ICC = 0.982) [21], the French FASH (ICC = 0.997) [22], the Spanish FASH (ICC = 0.993) [23] and the Persian FASH (ICC = 0.997) [24]. Excellent reliability was observed.

The correlation between SC-FASH and domains of EILP, VAS, as well as SF-36, were in accordance with the initial hypothesis: SC-FASH exhibited strong correlations with function-related questionnaires but showed relatively weaker correlations with psychologically oriented questionnaires. The associations between SC-FASH and domains of EILP, VAS were both very good in our study. One possible reason might be that during the SC-FASH assessment, we refer to the individual for acute hamstring injuries pain by dedicating a diagram to explain this in detail to the participants, which was which more similar to the tendency of the EILP and VAS assessments in terms of pain [25, 26]. It was also more specific. The association between SC-FASH and physical function was good. One possible reason for this was that physical function was similar to the pain experienced in acute hamstring injuries for evaluation purposes, as both affect limb function. The associations between SF-36 and role physical, bodily pain and general health were moderate in the present study [40]. One potential explanation for this discrepancy could be that the role physical, bodily pain and general health domains of the SF-36 were developed to assess function in daily living, but do not directly address pain from acute hamstring injuries. Additionally, the vitality, social function, role emotional and mental health subscales of the SF-36 were not associated with the SC-FASH. This phenomenon could be explained by the psychological impact of factors other than the physical environment [40]. The factorial validity of the SC-FASH questionnaire was confirmed by the strong support for the 2-factor solution. In the study, only minor adjustments were made for the cultural differences between China and the West. All questions were comprehensible to the participants and had adequate validity as the original FASH.

The present study demonstrated the validity of the SC-FASH in distinguishing individuals with acute hamstring injuries from those affected by other factors. These results are consistent with those previously reported for the FASH in other languages [20–24]. The questionnaire therefore would play an increasingly important role in supporting the huge amount of research, especially in quantifying patients’ functional status and analyzing data, as a growing focus on outpatient clinics for individuals with acute hamstring injuries caused by exercise.

There were several limitations in the present study. Firstly, although Simplified Chinese is the official language of China, the country is multi-ethnic and most minorities have their own language. It is therefore essential that the issue of national cultural differences be given due consideration. Secondly, the sample groups were limited, and no differentiation was made between patient groups, those engaging in regular physical activity groups, and those participating in professional sports groups. Future implementation will require collaboration with sports academies or fitness centers. Finally, follow-up with outpatients was challenging, as some patients failed to adhere to systematic treatment regimens, making it impossible to determine whether their condition has changed. Therefore, the SC-FASH response was not evaluated in the present study but will be in a subsequent study.

## Conclusions

The FASH has been successfully translated into Simplified Chinese and is a reliable and valid tool for physicians and researchers in mainland China to facilitate a more precise evaluation of not only the severity of the acute hamstring injury but also its progression until the individual is able to resume sporting activities.

## Data Availability

The datasets generated and/or analyzed during the current study are not publicly available due to some of the patient’s data regarding individual privacy, and according to the policy of our hospital, the data could not be shared with others without permission, but some are available from the corresponding author on reasonable request.

## Abbreviations

CIs: Confidence intervals
CITC: Corrected item-total correlation
HRQoL: Health-related quality of life
ICC: Intra-class correlation coefficient
FASH: The functional assessment scale for acute hamstring injuries
SC-FASH: The simplified Chinese version of the functional assessment scale for acute hamstring injuries
EILP: Exercise-induced leg pain questionnaire
VAS: Visual analogue scale
SF-36: Short form (36) health survey
SD: Standard deviation
BMI: Body mass index
SEM: Standard error of the mean
MDC: Minimum detectable change
EFA: Exploratory factorial analysis
CFA: Confirmatory factor analysis
